# Reconstruction of *Eriocheir sinensis* Protein–Protein Interaction Network Based on DGO-SVM Method

**DOI:** 10.3390/cimb46070436

**Published:** 2024-07-12

**Authors:** Tong Hao, Mingzhi Zhang, Zhentao Song, Yifei Gou, Bin Wang, Jinsheng Sun

**Affiliations:** Tianjin Key Laboratory of Animal and Plant Resistance, College of Life Sciences, Tianjin Normal University, Tianjin 300387, China; skyht@tjnu.edu.cn (T.H.); 18404964001@163.com (M.Z.); a953835912@gmail.com (Z.S.); ricardoyifie@hotmail.com (Y.G.)

**Keywords:** *Eriocheir sinensis*, aquatic crustacean, GO annotation, support vector machine, protein–protein interaction network

## Abstract

*Eriocheir sinensis* is an economically important aquatic animal. Its regulatory mechanisms underlying many biological processes are still vague due to the lack of systematic analysis tools. The protein–protein interaction network (PIN) is an important tool for the systematic analysis of regulatory mechanisms. In this work, a novel machine learning method, DGO-SVM, was applied to predict the protein–protein interaction (PPI) in *E. sinensis*, and its PIN was reconstructed. With the domain, biological process, molecular functions and subcellular locations of proteins as the features, DGO-SVM showed excellent performance in *Bombyx mori*, humans and five aquatic crustaceans, with 92–96% accuracy. With DGO-SVM, the PIN of *E. sinensis* was reconstructed, containing 14,703 proteins and 7,243,597 interactions, in which 35,604 interactions were associated with 566 novel proteins mainly involved in the response to exogenous stimuli, cellular macromolecular metabolism and regulation. The DGO-SVM demonstrated that the biological process, molecular functions and subcellular locations of proteins are significant factors for the precise prediction of PPIs. We reconstructed the largest PIN for *E. sinensis*, which provides a systematic tool for the regulatory mechanism analysis. Furthermore, the novel-protein-related PPIs in the PIN may provide important clues for the mechanism analysis of the underlying specific physiological processes in *E. sinensis*.

## 1. Background

*Eriocheir sinensis*, also known as Chinese mitten crab or river crab, is an aquatic species with great economic significance because of its delicious taste and nutritious flesh [1]. However, the systematic mechanisms of many biological processes in *E. sinensis*, including growth, development and molting, are still vague because the physiological processes in this species have not been systematically analyzed. The large-scale protein–protein interaction network (PIN) is an important tool for the systematic analysis of the mechanism of many biological processes. The reconstructed PINs have covered many organisms, from prokaryotes [2] to eukaryotes [3] and from monads [4] to humans [5]. The PINs have been applied to protein functional annotation, subsystem analysis, evolutionary analysis, hub protein analysis and human disease analysis [6]. The metabolic and regulatory mechanisms of the species-specific functions can be deeply explored if the related protein–protein interactions (PPIs) are included in the network, which is helpful to achieve insights into the unique phenotypes or characteristics of an organism, especially for non-model organisms. A large amount of PPIs can be detected through high-throughput experiments such as the yeast two-hybrid system and mass spectrometry [7,8]. PINs have been reconstructed based on the results of high-throughput experiments for *Homo sapiens* [9], *Saccharomyces cerevisiae* [4,10], *Drosophila melanogaster* [11] and *Caenorhabditis elegans* [12], providing valuable tools for the systematic study of the process of regulating growth and development in these organisms. However, most high-throughput experiments are prone to a large number of false positives and false negatives in the results [13,14,15]. Furthermore, sometimes, even when the same method is used to detect the PPIs, the results are still quite different [4,10]. In addition, compared with the actual number of PPIs in cells, relatively fewer PPIs are detected using experiments. Furthermore, due to time and cost constraints, high-throughput experimental data for analyzing PPIs in the cells of most organisms, such as *E. sinensis*, are lacking. These constraints limit the use of high-throughput experiments to reconstruct PINs and affect the quality of the reconstructed PINs.

Compared with high-throughput experiments, computational methods provide a more economical approach with a potential for wide application in PPI prediction. These methods are mainly based on homologous alignment and machine learning algorithms. The current PIN of *E. sinensis* was constructed based on homologous alignment with six model organisms [16]. However, homology-based reconstruction has some limitations. For example, this method may not capture species-specific PPIs, which seriously limits the analysis of biological processes unique to a particular species. In comparison, computational methods based on machine learning can detect more PPIs to gain a comprehensive understanding of PINs. Such methods classify unknown data based on its features. Researchers commonly use the sequence or structure (such as domain) of a protein as the feature to describe a protein and predict the PPIs [17]. Although the sequence-based method has yielded good results in some studies [18,19,20], its potential to accurately detect PPIs is limited by its focus on only sequence information, ignoring the information of structures. Protein domains, as the functional and structural units of proteins, are considered to have a strong influence on the interaction between proteins [21,22]. A study based on complete protein sequence showed that most proteins contain one or more domains, and protein domains can be regarded as an evolutionary unit of protein sequence [23]. Since this discovery, domain information has been used in the prediction of PPIs and has achieved good results, demonstrating that proteins containing a pair of positive interacting domains are likely to positively interact [24,25]. For example, Binny Priya et al. used a matrix-based method to predict PPI based on domain information with an overall accuracy of about 70% [26]. Hayashida et al. proposed a domain-based method using conditional random fields to predict PPIs and achieved an average accuracy of around 90% [27]. Singhal and Resat developed a method to score domain pairs first and then use these scores to predict whether a pair of proteins with the interacting domain pairs would interact [28]. All these research studies support the use of domain information for accurately predicting PPIs. However, protein interaction is a very complex process, and other factors besides protein domains can influence protein interactions, such as the biological process regulated by the protein, the molecular functions of a protein and the subcellular locations of a protein. Therefore, complementary methods are needed to validate the influence of domains in PPI prediction.

In this work, we first proposed a novel method, DGO-SVM, for comprehensive PPI prediction using domain, biological process, molecular function and subcellular location information of proteins. The performance and robustness of DGO-SVM were tested on the datasets of several organisms, including two model organisms, *Bombyx mori* and humans, and five aquatic crustaceans including *Penaeus vannamei*, *Armadillidium vulgare*, *Daphnia pulex*, *Daphnia magna* and *Tigriopus californicus*. Subsequently, the DGO-SVM method was used to predict the PPIs and reconstruct the PIN of *E. sinensis*. Our study showed that DGO-SVM is a valuable novel method for PPI prediction, and the PIN of *E. sinensis* provides a systematic tool for the comprehensive study of the mechanism of PPI-mediated biological activities in *E. sinensis* cells, especially physiological functions specific to this species.

## 2. Methods

### 2.1. Collection of PPI, Protein Sequence and Domain Dataset

**Figure 1:**
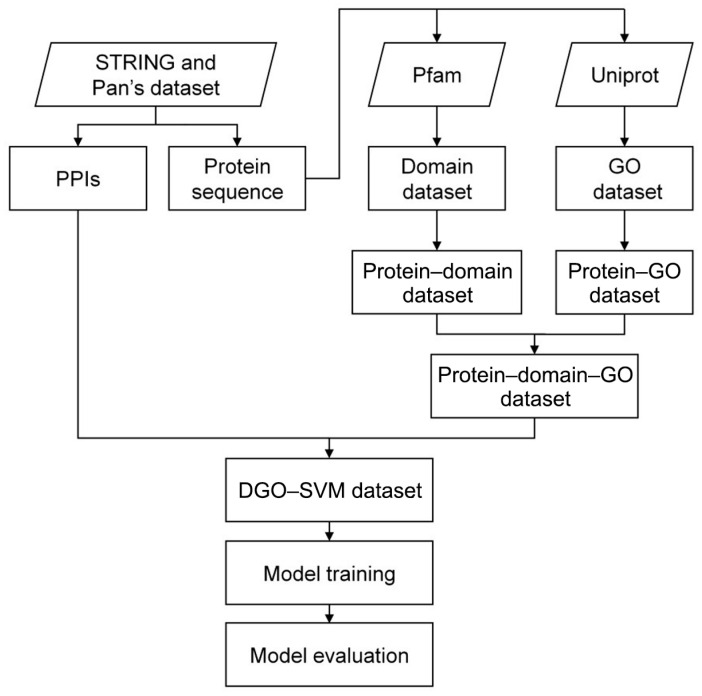
Flow chart of DGO-SVM method.

The flow chart of the DGO-SVM method is shown in Figure 1. *B. mori*, human and five aquatic crustaceans, including *P. vannamei*, *A. vulgare*, *D. pulex*, *D. magna* and *T. californicus*, were used to construct the DGO-SVM model and test its robustness. The PPIs and protein sequences of *B. mori* and the five aquatic crustaceans were collected from STRING database (https://cn.string-db.org, version 11.5, released on 12 August 2021). The PPIs and protein sequences of humans were directly obtained from Pan’s dataset [29].

The domain information of proteins was downloaded from the Pfam database (https://pfam.xfam.org, version 33.4, released on 24 March 2021) and aligned to the sequence of proteins to obtain the protein–domain relationship with the HMMER tool (version release 3.3.2, http://www.hmmer.org, accessed on 22 May 2021). The alignment results are stored as protein–domain dataset.

### 2.2. Collection of GO Dataset

The GO annotations of proteins in *B. mori*, humans and the five aquatic crustaceans were directly downloaded from the Uniprot database (https://www.uniprot.org, accessed on 25 June 2021). GO entries are organized into a tree structure. The farther away from the root of the tree, the more detailed the GO comments will be, and the more GO entries in the same level. In PPI prediction, more detailed GO entries do not mean more accurate prediction results. Therefore, to determine the optimal GO level for PPI prediction, GO entries were traced back through the tree structure, and the different GO levels were divided. The tree structure of the GO entries was obtained from the GO.OBO file (version 1.4, release monthly, downloaded on 29 November 2021) downloaded from the Gene Ontology (http://geneontology.org, accessed on 29 November 2021). The relationship of children and parental nodes was described as ‘is a’, ‘is part of’ and ‘regulates’ in the file. The Python program was used to trace each GO entry to the root of the tree and then the GO annotation of each protein was replaced by its related GO entries at a certain level. Finally, the GO entries in levels 1–6 were stored as six protein-GO datasets, with level 1 being the first level below the root of the tree, and the quantity of GO entries increasing with the increasing level.

### 2.3. Generation of Negative PPIs

Negative PPI data of *B. mori* and the five aquatic crustaceans were randomly generated, as PPIs collected from the STRING database can only be used as positive PPIs. The function ‘random.randint’ was used to randomly generate two integers and the corresponding numbered proteins were extracted in the protein dataset. Then, this protein pair was checked if it existed in the whole positive PPI dataset downloaded from the STRING database and if it had already existed in the negative PPI dataset. If one of the two situations appeared, this protein pair was abandoned and two other proteins were regenerated randomly. If not, this protein pair was considered a negative PPI. STRING gives evidence scores for an interaction from different channels, including neighborhood, fusion, co-occurrence, co-expression, experimental, database and text-mining. The combined score, in a range of 0–1000, is computed by combining the probabilities from the different evidence channels and is corrected for the probability of randomly observing an interaction, which to some extent reflects the interaction possibility of two proteins. Therefore, to learn the interaction rules more accurately, the PPIs with a combined score > 900 were filtered out as the positive PPIs and used for model training and testing. The generation of negative PPIs was repeated until the number of negative PPIs was equal to the positive PPIs. Finally, the negative PPIs and positive PPIs were used for data training and testing to ensure the reliability of the dataset. The negative PPIs in humans were directly obtained from Pan’s dataset [29].

### 2.4. Generation of the Dataset for PPI Prediction

To obtain the training dataset and testing dataset, we first integrated the corresponding protein–GO dataset and protein–domain dataset to form the feature vectors of proteins. Domains and GO annotations were used as the features of each protein. All the non-repeated domains and GO annotations in the protein–GO dataset and protein–domain dataset were combined as independent features, and 1 or 0 was used to indicate whether the domain or GO entry exists in a protein. A feature vector containing the information of domains and GO annotations was constructed for each protein.

**Figure 2:**
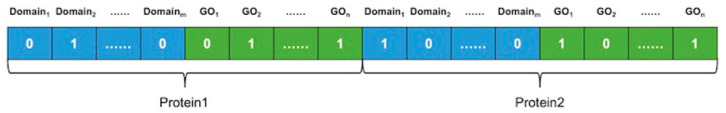
The feature representation of PPIs.

Subsequently, the feature vectors of the two proteins in a protein interaction were connected to form a vector of the PPI (Figure 2). In order to improve the performance of features and better explore the potential vector patterns for the PPIs, the two protein vectors were connected in a different order to generate two mirrored interacting vectors. For example, if protein 1 and protein 2 with vectors V1 and V2, respectively, were in an interaction, two vectors of interactions were generated for this PPI: V1–V2 and V2–V1. The vectors were generated for both positive and negative PPIs.

**Figure 3:**
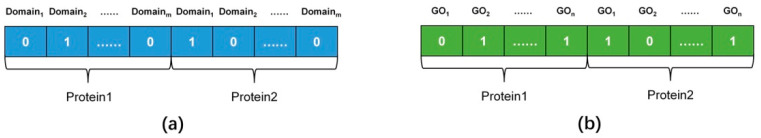
The feature representation of PPIs taking protein domains and GO annotations as features, respectively. (**a**) The feature presentation of PPIs taking protein domains as features. (**b**) The feature presentation of PPIs taking GO annotations as features.

We also performed testing with only protein domains or GO annotations as features. The feature vectors of the proteins were composed of only domains or GO annotations. The feature vectors for the PPIs are shown in Figure 3.

After generating feature vectors, the PPIs in the dataset were randomly shuffled to ensure that positive PPIs and negative PPIs were randomly distributed, which can prevent overfitting of the SVM model. Finally, the dataset was divided into five groups, with the split ratio for the test and training datasets as 4:1 for the convenience of the following five-fold cross-validation.

### 2.5. Classification

SVM has been widely used as the core algorithm for the prediction of PPIs in biological studies, with good performance [30,31,32,33,34]. Kernel selection is a critical factor that significantly influences prediction results. Four commonly used SVM kernels—Linear, Polynomial, Sigmoid and RBF—were tested on the *B. mori* dataset to evaluate the performance of DGO-SVM with various kernels. Grid search was employed to optimize the parameters C and γ for each kernel. The Polynomial kernel used a default degree value of 3. The default value of Coef0 was used for Polynomial and Sigmoid kernels. The optimal values of C and γ in DGO-SVM are different for different types of organisms. The optimal values for *B. mori* are C = 8, γ = 0.05; for humans, they are C = 36, γ = 0.01; and for the aquatic crustaceans and *E. sinensis*, they are C = 8, γ = 0.055. The RBF kernel was finally set as the kernel function of DGO-SVM, which is regarded as the most efficient high-dimensional mapping function. The base function of SVM was defined as follows:

(1) $$min\frac{1}{2}{\left|\left|\omega \right|\right|}^{2}+C\sum_{i=1}^{n}\zeta_{i}$$

where hyperparameter C trades off the correct classification of training examples against the maximization of the decision function’s margin. When the value of C is large, the decision margin will be smaller, encouraging the SVM to better classify all training points correctly. In contrast, when the value of C is smaller, the decision margin will be larger, and the SVM is more likely to make a simpler decision. The RBF kernel was defined as follows:

(2) $$K\left(x,y\right)=exp\left(-\gamma {\left|\left|x-y\right|\right|}^{2}\right)$$

where hyperparameter γ defines how far the influence of a single training example reaches. A low γ value means that each single training example has a high influence on the decision of the model and a high value means the influence is low.

As the whole dataset has already been shuffled and divided into five groups, the five-fold cross-validation was used to evaluate the stability of the method, which is a commonly used method for the evaluation of the machine learning model in many research studies [35,36,37]. Each of the five datasets was used as the training dataset in turn, with the remaining four datasets as test datasets, so validations proceeded to evaluate the model five times. The SVM model was built with the scikit-learn toolkit: version release 1.1 (https://scikit-learn.org/stable/, accessed on 10 January 2022) that was developed based on libsvm.

### 2.6. Evaluation of DGO-SVM Method

Seven indicators were used to evaluate the prediction performance of the DGO-SVM method, including accuracy (Acc), sensitivity (Sn), specificity (Spe), precision (Pre), F1 score, Area Under Curve (AUC) and Matthews correlation coefficient (MCC). Acc, Sn, Spe and Pre are basic and intuitive indicators for evaluation. Acc represents the proportion of correctly predicted data in the overall data, Sn represents the proportion of correctly predicted results in positive results predicted by the model, Spe represents the proportion of correctly predicted results in negative results predicted by the model and Pre represents the proportion of predicted positive results to actual positive numbers. F1, AUC and MCC are comprehensive evaluation indicators that attempt to convey the quality of predictions with a single value. F1 is the harmonic mean of Sn and Pre, which is high only when both Sn and Pre are high. MCC is considered an unbiased version of F1. A MCC value close to 1 indicates very accurate prediction, while a value close to 0 indicates that prediction is not better than random guessing. There is another parameter called false positive rate (FPR), which is the proportion of errors in the model’s predicted positive results. A ROC curve is formed by drawing curves with Sn and FPR as the vertical and horizontal axes, respectively. The AUC value is the area covered by the ROC curve, ranging from 0 to 1. The larger AUC reflects the better classification performance of the algorithm. The mathematical descriptions of these parameters were defined as follows:

(3) $$Acc=\frac{TP+TN}{TP+FP+TN+FN}$$

(4) $$Sn=\frac{TP}{TP+FN}$$

(5) $$Spe=\frac{TN}{FP+TN}$$

(6) $$Pre=\frac{TP}{TP+FP}$$

(7) $$F1=\frac{2TP}{2TP+FP+FN}$$

(8) $$AUC=\frac{\sum_{i\in positiveClass}{rank}_{i}-\frac{\left(TP+TN\right)\left(1+TP+TN\right)}{2}}{\left(TP+TN\right)\times \left(FP+FN\right)}$$

(9) $$MCC=\frac{TP\times TN-FP\times FN}{\sqrt{\left(TP+TN\right)\times \left(TN+FP\right)\times \left(TP+FP\right)\times \left(TN+FN\right)}}$$

where TP (the true positive value) is the number of positive PPIs predicted correctly, TN (the true negative value) is the number of negative PPIs predicted correctly and FN (the false negative value) and FP (the false positive value) are the number of positive and negative PPIs predicted incorrectly, respectively.

### 2.7. Comparison of DGO-SVM with Other Machine Learning Methods

The performance of Logistic Regression (LR) and Random Forest (RF) was evaluated with the same human dataset for DGO-SVM. In the test of LR, the C value was set to the default of 1.0. L2 regularization (penalty = ‘l2’) was used because there is no clear distinction between domains and GO as features, and L2 encourages the classifier to use all features. The solver used was liblinear, which performs well in binary classification problems [38]. In the DT test, the parameter n_estimators was set to 200, and all other parameters were set to the default values of the RandomForestClassifier in Python’s sklearn.ensemble module.

### 2.8. Prediction of PPIs in E. sinensis

The protein information of *E. sinensis*, including the sequences and GO annotations, was obtained from its genome sequencing data from the BioProject database under the accession number PRJNA555707 [39]. The protein sequences were aligned with the domains from the PFAM database to obtain the protein–domain relationship. The GO annotations were backtracked to level 4 and used as features for the SVM model. Candidate PPIs were generated based on the alignment and combination of all the proteins in *E. sinensis* without generating the negative PPIs or shuffling the dataset. The DGO-SVM was applied to the *E. sinensis* dataset with the values of C and γ set as the optimized C and γ values for the combination dataset of the five aquatic crustaceans. The GO enrichment of the novel proteins in *E. sinensis* PIN was analyzed with TBtools software (version 2.041, https://github.com/CJ-Chen/TBtools-II/releases, accessed on 3 November 2023) [40].

## 3. Results

### 3.1. Dataset of B. mori

In total, 10,613 protein sequences and 3,258,118 positive PPIs of *B. mori* were obtained from the STRING database. The domain dataset of *B. mori* was downloaded from the Pfam database and 5591 domains were identified in *B. mori* by aligning the protein sequences of *B. mori* to all the domain sequences from the Pfam database. A total of 2768 GO annotations of *B. mori* were collected from the Uniprot database. Among the PPIs obtained from the STRING database, 52,764 positive PPIs with scores > 900 were screened out. The same number of negative PPIs was randomly generated for *B. mori* (Supplementary File S1). Further, as described in the Section 2, two protein vectors were connected in two different orders to generate 105,528 reverse PPIs. Therefore, including the reverse PPIs, the whole dataset contained 211,056 PPI relationships (Table 1).

### 3.2. GO Backtracking

By backtracking through the tree structure of GO annotations described in the OBO file, six different levels (level 1 to level 6) of GO annotations were finally obtained. The number of GO entries included in each level in the OBO file and *B. mori* is shown in Table 2. The GO annotations in the high level, which is closer to the root of the tree structure, covered all the annotations in the lower level. Therefore, the GO entries in one level rather than all the GO entries in the OBO file were separately used as features of the proteins to avoid feature duplication. The lower the GO level, the more detailed the annotations become, resulting in a greater quantity of GO entries at that level. The GO annotations in each level are listed in Supplementary File S2.

### 3.3. Determination of GO Level Used for PPI Prediction

The different level of GO annotations was shown to influence the prediction results when GO was taken as part of the features of proteins. As the accuracy of the prediction result may not increase with the details of GO entries, the GO level most suitable for the PPI prediction needs to be further determined. Therefore, for each level of GO annotations, a dataset was constructed for the PPI prediction. Furthermore, the original GO annotations of *B. mori* proteins obtained directly from the Uniprot database, which were not traced back through the tree structure, were used for testing. The features of each protein were composed of domains and GO annotations, as described in the Section 2. For example, in level 4, there were 990 GO annotations and 5591 domains in *B. mori*. Therefore, the dimension of the feature vector for each protein was 6581 (5591 + 990). As the feature vector of a PPI was the combination of two proteins, its dimension was 13,162, that is, each PPI vector constructed based on the level 4 GO annotations contained 13,162 features.

**Figure 4:**
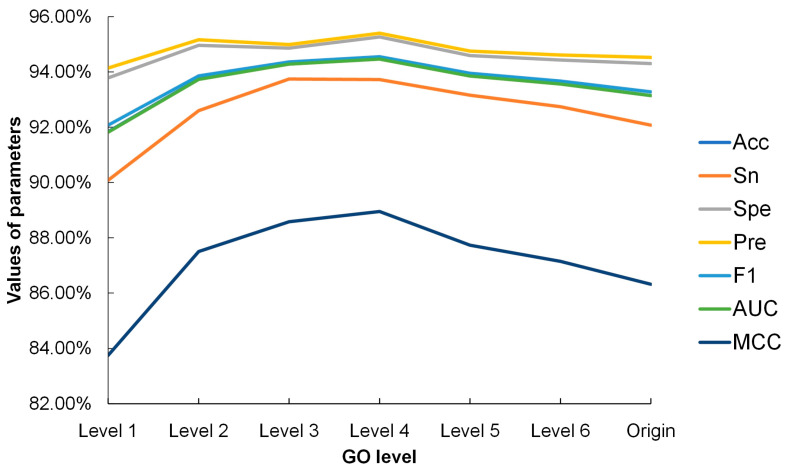
The results of PPI prediction on *B. mori* dataset with different GO levels. Acc stands for accuracy; Sn stands for sensitivity; Spe stands for specificity; Pre stands for precision; F1 stands for F1 score; AUC stands for Area Under Curve; MCC stands for Matthews correlation coefficient. The lines corresponding to Acc and AUC overlapped in the graph due to their values being too close to each other.

Seven datasets with different GO levels, including levels 1–6 and origin GOs, were separately imported into the SVM model for testing. Optimal C and γ parameters were determined using the grid search method for each dataset and used to fit the SVM model. The prediction performance of the model on each dataset is shown in Figure 4. The results show that all datasets achieved good prediction performances with over 90% accuracy. In particular, the accuracy rates for level 3 and level 4 were higher than 94%, which proved that the use of GO annotations as features of proteins enhances the accuracy of PPI prediction. Interestingly, the accuracy of the original GO annotations of 93.14% was only higher than level 1. This result further demonstrated that original GO annotations may not be the optimal features for PPI prediction, suggesting the need to find the best GO level for the prediction. As shown in Figure 4, the dataset with level 4 GO annotations showed the best performance with 94.47% accuracy. Therefore, level 4 GO annotations were finally used as the GO features in the DGO-SVM method. Using the backtracked GO annotations also reduced the dimension of features, saving the runtime of classification.

### 3.4. Robustness Test of DGO-SVM Method on B. mori and Human Datasets

Robustness is an important evaluation indicator of machine learning models, mainly used to test whether the model’s performance is stable when facing certain changes. The level of robustness directly determines the generalization ability of machine learning models. Robustness in this work is considered the stability of multiple predictions (five-fold validation) on the same dataset and the stability of the prediction results on different datasets. It was considered here that a less than 1% difference between multiple predictions on the same dataset (test of the model on a single organism) and less than 5% difference between predictions on different datasets (test on different organisms of interest) was robust. This indicator requires the model to fluctuate within a small range when facing changes.

**Figure 5:**
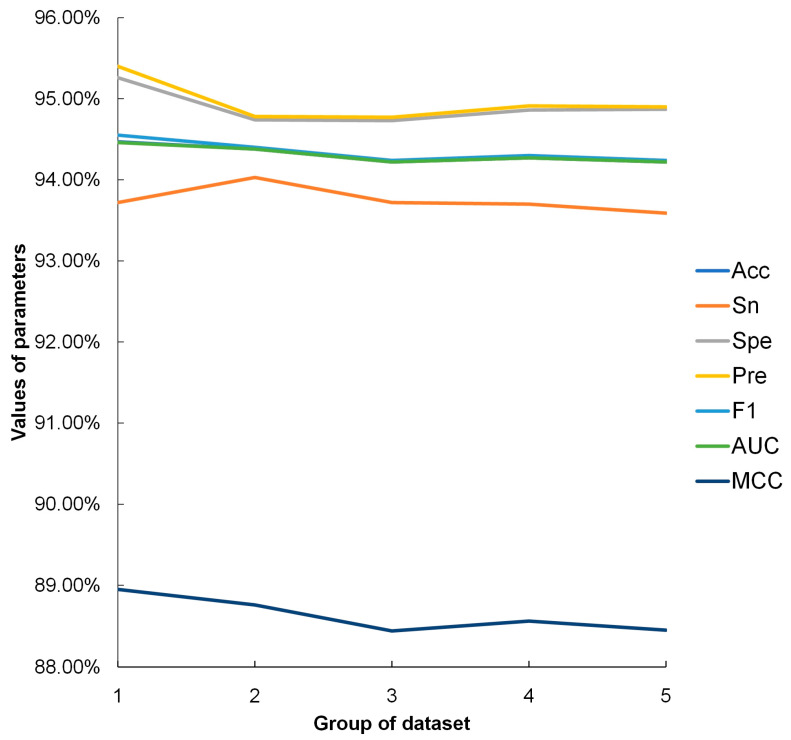
The results of five-fold validation for *B. mori* dataset with level 4 GO. Acc stands for accuracy; Sn stands for sensitivity; Spe stands for specificity; Pre stands for precision; F1 stands for F1 score; AUC stands for Area Under Curve; MCC stands for Matthews correlation coefficient. The lines corresponding to Acc and AUC overlapped in the graph due to their values being too close to each other.

To test the robustness of the DGO-SVM method, the *B. mori* dataset based on level 4 GO annotations was equally divided into five groups and used for five-fold cross-validation. The optimized values of C and γ were set as 8 and 0.05 and searched for using the grid search method. The prediction results are shown in Figure 5. The highest and average prediction accuracies of level 4 GO annotations were 94.47% and 94.31%, respectively. There was no obvious fluctuation (<1%) in the whole curve, suggesting that the performance of the DGO-SVM method in predicting PPIs was excellent.

To further test the robustness of the DGO-SVM method on other species, it was applied to Pan’s dataset [29], which was constructed with human protein interactome and has been widely used in PPI studies [41,42]. In total, 10,133 protein sequence data and 73,110 protein–protein interaction data were contained in Pan’s dataset, including 36,630 positive and 36,480 negative PPIs. Applying the same method used to build the *B. mori* dataset, the human dataset was constructed with domains and level 4 GO annotations as the features of proteins. Subsequently, the dataset was equally divided into five groups for five-fold cross-validation to further evaluate the robustness of the DGO-SVM method on the human dataset. The prediction results based on Pan’s dataset are shown in Table 3. All the evaluation indicators were higher than 90% and the average accuracy was 95.52%, which is 1.21% higher than that of *B. mori*. It indicates that the good performance of DGO-SVM was not influenced by the type of organism. The fluctuation of the results was also less than 1%. Considering the stability of the DGO-SVM method, it can be widely applied to any organism with genome-wide annotation data.

As shown in Table 3, the DGO-SVM method achieved an excellent performance on Pan’s dataset with the highest and average prediction accuracy of 95.65% and 95.52%, respectively. Further, the model showed a high accuracy in classifying almost all PPIs with Sn, Spe and Pre of 97.73%, 93.47% and 93.27%, respectively. The F1 and MCC are two comprehensive and better indicators of the overall performance of a model. The average F1 and MCC values were 95.45% and 91.14%, respectively, which is comparable to the other indicators and proves that the model performs well in comprehensive aspects. The prediction results on *B. mori* and human datasets showed that both the domains and GO annotations are important features for protein interactions.

### 3.5. Performance of Difference Kernels for SVM Model

The selection of kernels in the SVM model significantly influences the prediction results. Commonly used kernels include Linear, Polynomial, Sigmoid and RBF. Linear and low-degree Polynomial kernels tend to solve linearly separable problems, while high-degree Polynomial, Sigmoid and RBF tend to solve non-linearly separable problems. In the selection of kernels, the data characteristics and quantity are usually considered. If the data are linearly separable in high-dimensional space, a Linear kernel might be a good choice. If the data have complex non-linear relationships in low-dimensional space, Polynomial or RBF kernels are commonly used. Additionally, from the perspective of the number of samples and features, if the sample size is close to the number of features, the Linear kernel is used. If the number of features is less than the number of samples, RBF is more suitable. For PPI prediction, the features of proteins, such as sequences, domains and GO annotations, are usually not linearly separable, and the number of protein features is typically less than the number of interaction relationships (i.e., the number of samples). Therefore, after comprehensive consideration, RBF was chosen as the kernel function in the preliminary experiments. However, to further determine the most suitable kernel function, the performance of the four kernels was tested using the B. mori dataset, with the results shown in Table 4. The results indicate that the accuracies of the four kernels are RBF > Polynomial > Linear > Sigmoid. This result further confirms the non-linear characteristic of PPI data. Therefore, RBF was finally chosen as the kernel for subsequent experiments.

### 3.6. Influence of Protein Domains and GO Annotations on PPIs

DGO-SVM uses two kinds of features for PPI prediction: protein domains and GO annotations. To evaluate the influence of the two kinds of features on the PPI predictions, the PPIs of humans and *B. mori* were predicted with only protein domains or GO annotations as features. The results are shown in Table 5. In general, there was no significant difference in the values of evaluation indicators based on GO annotations and domains. However, compared with domain-based indicators, most GO-based indicators were slightly higher in humans and slightly lower in *B. mori*. The results indicate that, as model features, the GO annotations perform as well as the domains. The prediction accuracy of DGO-SVM on *B. mori* and human datasets was 94.31% and 95.52%, which indicated that combining protein domains and GO annotations as features could further improve the accuracy of prediction.

### 3.7. Comparison with Other Methods

To demonstrate the high prediction performance of the DGO-SVM method, we compared it with other studies. For a more accurate comparison, five different methods that have also used Pan’s dataset and SVM classifier were selected, including LDA-SVM, SP-SVM, PseAAC_SVM [29] and the methods of Goktepe et al. [43] and Zhang et al. [44]. LDA-SVM, SP-SVM, PseAAC_SVM and Goktepe’s methods predict the PPIs based on the protein sequences, including latent topic sequential feature [29], extending the signature descriptor to protein pairs [45], pseudo amino acid composition sequential feature [46] and the weighted skip-sequential conjoint triads [43], respectively. Zhang’s method predicted the PPIs based on the protein domains [44]. The results of the comparison of these five methods are shown in Table 6. The DGO-SVM method outperformed all other methods in the evaluation indicators, including Acc, Sn, Spe and MCC. Notably, the DGO-SVM method had the best performance in Sn, demonstrating its ability to accurately detect positive PPIs. In addition, since no GO annotation was used in any of the five other methods, the biological process, molecular function and subcellular location information of proteins represented by GO annotations are quite important for the accurate prediction of PPIs.

In addition to SVM, other machine learning methods can also be applied to PPI prediction, such as Logistic Regression (LR), Decision Trees (DT), Random Forest (RF), etc. Among structure-based prediction methods, SVM, LR and RF are commonly used [47]. Therefore, we utilized Pan’s dataset, employing the same features used in DGO-SVM, to predict PPIs with LR and RF algorithms and compared their prediction performance with that of DGO-SVM. The results are shown in Table 7. The results indicate that the accuracies using the LR and RF algorithms are both higher than 93% but slightly lower than that of DGO-SVM. This suggests that the domain and GO features are robust across various machine learning methods, with DGO-SVM performing the best among these methods.

### 3.8. Performance of DGO-SVM on Aquatic Crustaceans

As with most aquatic crustaceans, *E. sinensis* is not a model organism. Therefore, to test the availability of the DGO-SVM in aquatic crustaceans, it was applied to five aquatic crustaceans, including *P. vannamei*, *A. vulgare*, *D. pulex*, *D. magna* and *T. californicus*. The positive and negative PPIs of the five aquatic crustaceans are provided in Supplementary File S1. The statistics of the datasets for the five aquatic crustaceans and the prediction results are shown in Table 8 and Table 9, respectively. The prediction accuracy of DGO-SVM for all five species was higher than 93%. Notably, for *D. pulex*, most of the evaluation indicators were above 96%. The fluctuation of prediction results between different organisms was less than 5%. These results demonstrated the excellent performance of DGO-SVM in predicting the PPIs of aquatic crustaceans. In addition, the model’s Spe and Pre were more prominent compared with the other evaluation indicators, suggesting that DGO-SVM can efficiently identify negative PPIs in crustaceans. The predicted results of PPIs among the aquatic crustaceans further demonstrate the good stability of DGO-SVM and its wide applicability in different species.

Furthermore, to evaluate the availability of DGO-SVM in larger datasets, it was applied to the combination dataset of the five aquatic crustaceans with 2,591,892 PPIs. The prediction accuracy of DGO-SVM on the integrated dataset was 92.26%, with the optimized values of C and γ of 8 and 0.055 using the grid search method. The Sn, Spe, Pre, F1, MCC and AUC were 91.79%, 92.76%, 92.84%, 92.31%, 84.54% and 92.27%, respectively. These results further confirmed the availability of DGO-SVM in larger datasets.

### 3.9. Reconstruction of E. sinensis PIN

**Figure 6:**
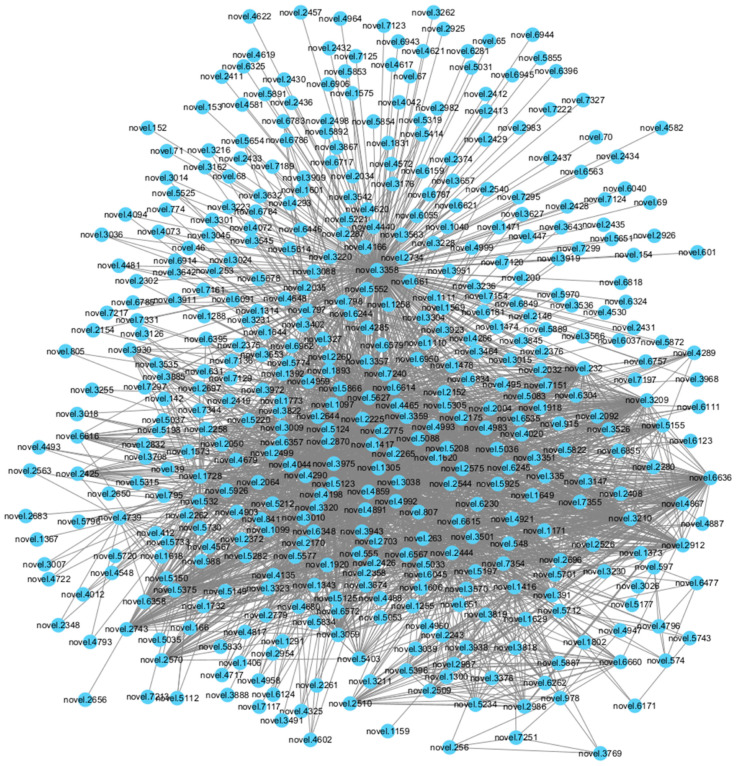
The PINs related to novel proteins with scores > 0.9 in *E. sinensis*.

The DGO-SVM was applied to *E. sinensis* to predict the positive PPIs for reconstructing the PIN. Candidate PPIs of *E. sinensis* were generated according to its genome annotation [39]. A total of 14,703 proteins were identified in *E. sinensis*, and after alignment and combination, 108,096,456 candidate PPIs were generated for these proteins for prediction. These proteins were associated with 6183 domains and 2300 original GO annotations. The number of level 4 GO annotations was 856 after tracing back through the tree structure. Therefore, the dimension of the feature for each candidate’s PPI was 7039 (6138 + 856). The DGO-SVM was then applied to the *E. sinensis* dataset. The values of C and γ were set as 8 and 0.055, which were the optimized values for the combination dataset of the five aquatic crustaceans. Finally, 7,243,597 positive PPIs were predicted. Based on the prediction results, we reconstructed the PIN of *E. sinensis,* comprising 14,703 proteins and 7,243,597 PPIs (Supplementary File S3), including 566 novel proteins from genome annotation of *E. sinensis* and 35,604 related PPIs (Supplementary File S4). During the prediction, the DGO-SVM model calculated the score of each candidate’s PPI and judged the classification results according to the score. The candidate PPIs with scores larger than 0.5 were predicted to be positive PPIs. The score distribution of the positive PPIs and novel protein-related PPIs in *E. sinensis* are shown in Table 10. There were 947,893 positive PPIs with interaction scores greater than 0.9, which included 2938 novel protein-related PPIs (Figure 6).

**Figure 7:**
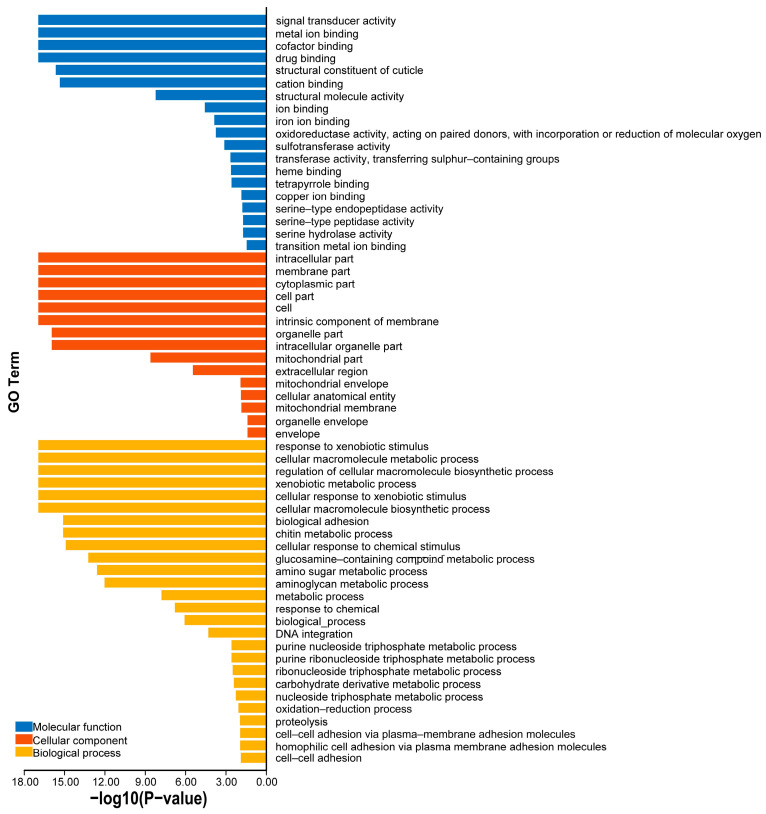
GO enrichment analysis of novel proteins in *E. sinensis*.

The GO enrichment of the 566 novel proteins in the *E. sinensis* PIN was further analyzed, and the results are shown in Figure 7. In the molecular function component, the identified proteins were mainly associated with signal transduction activity, metal ion binding and cofactor binding. In subcellular locations, the proteins were enriched in the intracellular part, the membrane part and the cytoplasmic part. In biological processes, the proteins are centrally distributed in response to exogenous stimuli, cellular macromolecule metabolic processes and regulatory processes of cellular macromolecule biosynthesis.

## 4. Discussion

### 4.1. Features of DGO-SVM Method

Feature selection plays a crucial role in the prediction outcomes of machine learning models. The SVM has been widely used and shows good performance in predicting PPIs owing to the fact that PPI prediction is considered a binary classification problem regarding pairs of proteins interacting or not. Therefore, SVM is commonly applied in PPI prediction whereby protein sequences and structures are mainly used as the features. However, in the prediction of PPIs, the function and location information of proteins are often ignored. Moreover, function information is commonly applied in protein function prediction because the interacting proteins are usually considered to have similar functions, indicating that the protein functions are strongly related to the protein interactions [4,48]. Furthermore, it has been demonstrated that protein interactions often occur between a pair of proteins that are located in the same or neighboring cellular regions. For instance, two interacting proteins may be found in the cytoplasm or on the cellular membranes and extracellular space. These findings suggest that protein localization is closely linked to their ability to interact with other proteins. In this study, the information of protein functions and locations, which is described as the GO annotations, was used as features to construct the SVM model as well as the structure information (domain of protein). The GO annotations were added as features in DGO-SVM to improve the PPI prediction performance of the model. The method was then verified on the *B. mori*, human and five aquatic crustacean datasets. The GO annotations for the *B. mori*, human and five aquatic crustaceans were obtained from the Uniprot database, which were annotated by the Gene Ontology Annotation (GOA) project completed by EMBL-EBI [49]. The GO annotations of *E. sinensis* were obtained by matching the GO ID from the GOA database to the interPro entry of proteins predicted using InterProScan70 (v5.31) software. Studies have shown that the methods used for processing features can significantly affect the accuracy of predictions. For instance, some structure-based SVM models that utilized different feature processing methods demonstrated significantly different performance levels, with accuracy ranging from 70% to 93%. [26,27,28]. Similarly, the accuracy of the sequence-based SVM models with different feature-processing methods varies in the range of 70% to 85% [18,19,21,50]. For GO annotations, different proteins are annotated at various detail levels; i.e., not all proteins are annotated at the same detail. Therefore, when using GO as features, the GO annotations were initially mapped to specific hierarchical levels based on the structure of GO terms. Subsequently, annotations from a single level were utilized as features to ensure that the GO features for all proteins were standardized to the same level. The GO annotations were tested individually at each level to identify the most suitable level for PPI prediction. Ultimately, the level 4 GO annotations exhibited the highest accuracy and were employed as GO features in the DGO-SVM.

Different organisms might have different coverage in GO, with the human and *B. mori* datasets expected to be the most complete. These differences could explain variations in prediction accuracy across species. Among the five aquatic crustaceans studied, four showed lower prediction accuracy compared to humans and *B. mori*. Since none of these aquatic crustaceans are model organisms, we chose to enhance model accuracy by testing features and kernels using the *B. mori* dataset, which is a model organism belonging to the arthropod phylum, similar to aquatic crustaceans. Additionally, *B. mori*’s large genome, comprising more than 20,000 genes, also enabled the assessment of the DGO-SVM method’s performance on a large-scale dataset.

Moreover, given the predominant focus of PPI prediction research on the human dataset, the DGO-SVM was also applied to humans (Pan’s dataset) for comparative analysis with other methods and to test its robustness across species. The DGO-SVM model demonstrated outstanding performance on the human dataset, achieving a high prediction accuracy of 95.65%. Following confirmation of features and kernels, the model was further evaluated on datasets from five aquatic crustaceans to assess its applicability in this group and achieved prediction accuracies exceeding 93%. The strong performance across *B. mori*, human and aquatic crustacean datasets indicates that the DGO-SVM can be effectively applied to various species with comprehensive genome annotations. After rigorous validation, the DGO-SVM method was used to predict PPIs in *E. sinensis*, resulting in predicted PPIs of high reliability.

In this study, PPIs from the STRING database served as the ‘ground truth’ for model training and testing. All interactions in STRING are assessed and scored using a ‘combined score’ ranging from 0 to 1000. This score reflects STRING’s confidence in the biological relevance of each interaction, computed by integrating probabilities from various evidence channels, including neighborhood, fusion, co-occurrence, co-expression, experimental data, databases and text mining. For this research, only interactions with a combined score > 900 were selected as positive PPIs for model training and testing to ensure the reliability of the prediction results. Consequently, interactions in STRING based solely on predictions, which commonly has lower scores, were excluded from the dataset. This approach ensured that the predicted PPIs overlapped significantly with high-confidence interactions in the STRING database, as evidenced by the high scores of positive PPIs in both the training and testing datasets.

In addition, GO annotations have the potential to be used separately as a good feature for PPI prediction, with data showing a 93.50% accuracy in the human dataset, exceeding the accuracy of ordinary protein domains (93.39%). This suggests that biological processes, molecular functions and subcellular locations of proteins are also important factors that influence the interaction between proteins and can thus be used in the computational prediction of PPIs. In previous studies, the GO features of proteins were commonly overlooked when predicting PPIs. However, the utilization of both GO annotations and protein domains has been shown to yield more accurate PPI predictions, as demonstrated by the DGO-SVM model. This could be because features based solely on Pfam domains might overlook certain information, such as interactions between domains and linear motifs, while GO annotations could effectively complement these limitations of domain features. Interestingly, the good performance of DGO-SVM provides a new perspective on the investigations on protein interactions. Based on the prediction results of this work, more detailed information should be considered in future studies on PPIs, besides the sequence or structures of proteins. Some of the information that should be considered includes the biological process, molecular function and subcellular locations.

### 4.2. PIN of E. sinensis

To reconstruct the published PIN of *E. sinensis*, a homologous alignment was performed with six model organisms. The resulting network contained 8225 proteins and 148,524 interactions [16]. In comparison, the PIN reconstructed in this work comprised 14,703 proteins and 7,243,597 PPIs. This large difference between the two PINs suggests that the homologous alignment method may lose substantial information in PPI prediction. More importantly, compared with the homologous alignment-based PIN, the PIN reconstructed with DGO-SVM contains 566 *E. sinensis*-specific proteins and 35,604 related PPIs.

From the scale of the five aquatic crustaceans obtained from the STRING database, the PIN with tens of thousands of proteins usually contains several millions of PPIs. For example, for *D. pulex*, its PIN comprises 15,113 proteins and 8,430,684 PPIs. It is worth highlighting that among the five studied aquatic crustaceans, *D. pulex* exhibits the highest prediction accuracy for PPIs, reaching 96.51%. Furthermore, the size of the *E. sinensis* PIN reconstructed using the DGO-SVM method is consistent with the network sizes observed in the other five aquatic crustaceans. This finding provides additional evidence that supports the validity of the reconstructed *E. sinensis* PIN.

The present PIN of *E. sinensis* was constructed based on the homologous alignment with six model organisms, including *H. sapiens*, *D. melanogaster*, *C. elegans*, *Rattus norvegicus*, *Mus musculus* and *S. cerevisiae*. The PIN includes 8225 proteins and 148,524 PPIs, whose scale is much smaller than the PIN with a score > 0.9 constructed with DGO-SVM. Most importantly, the species-specific PPIs cannot be predicted with the homologous alignment method, while in the PIN constructed in this work, the interactions among 566 novel proteins, which specifically exist in *E. sinensis*, were successfully captured.

The novel proteins in the *E. sinensis* PIN were mainly associated with processes such as response to exogenous stimuli, cellular macromolecule metabolic processes and regulatory processes of cellular macromolecule biosynthesis. This indicates that *E. sinensis* may have a unique response mechanism to exogenous stimuli, and the metabolism and regulation of some cellular macromolecules may different from other organisms. These novel proteins may be closely related to some special physiological phenomena in *E. sinensis*, such as molting, limb regeneration and development suspending, which are regulated by the macromolecular hormones. The PPIs associated with the novel proteins can provide valuable insights for analyzing the mechanisms underlying these physiological phenomena.

## 5. Conclusions

PPIs harbor important information for understanding the mechanisms associated with various biological processes. A comprehensive and high-quality PIN of *E. sinensis* greatly assists the mechanism analysis for many physiological processes. Machine learning has become an important method for PPI prediction due to its completeness and continuously improving accuracy. In this work, a novel method DGO-SVM was first proposed for PPI prediction. The method utilizes the information of domains and backtracked GO annotations as features of proteins to construct a SVM model for classification. The prediction results of PPIs on *B. mori*, human and five aquatic crustaceans showed that DGO-SVM had a satisfactory performance on PPI prediction with a stable high accuracy. This result demonstrated that besides domains, the biological process, molecular function and subcellular location information of proteins, which is represented by GO annotations, can also improve the accuracy of PPI prediction. The separate testing of features demonstrated that the combination of domains and GO annotations improved the prediction accuracy compared to ordinary domains or GO annotations as features. The PIN reconstructed in this work using DGO-SVM is the most extensive network for *E. sinensis* to date, and the newly incorporated protein–protein interactions may offer significant insights for analyzing the mechanisms behind specific physiological phenomena in *E. sinensis*.

## Figures and Tables

**Table 1:** Statistic of *B. mori* dataset.

Protein	Domain	GO	Positive PPIs	Positive PPIs (Score > 900)	Negative PPIs	Total PPIs
10,613	5591	2768	3,258,118	52,764	52,764	211,056

**Table 2:** Number of GO entries in six GO levels.

GO Level	Number of GO Entries in OBO File	Number of GO Entries in *B. mori*
Level 1	3	3
Level 2	56	40
Level 3	1446	338
Level 4	4769	990
Level 5	9948	1694
Level 6	13,778	2370

**Table 3:** Results of five-fold cross-validation on Pan’s dataset.

Dataset	Acc	Sn	Spe	Pre	F1	AUC	MCC
	(%)						
Data 1	95.65	97.76	93.72	93.45	95.56	95.65	91.40
Data 2	95.30	97.69	93.12	92.83	95.20	95.31	90.72
Data 3	95.65	97.82	93.66	93.43	95.58	95.67	91.41
Data 4	95.44	97.68	93.34	93.21	95.39	95.47	90.98
Data 5	95.55	97.71	93.51	93.42	95.52	95.58	91.20

**Table 4:** Performance of different kernels in DGO-SVM.

Kernel	C	γ	Acc	Sn	Spe	Pre	F1	AUC	MCC
			(%)						
RBF	8	0.05	94.47	93.72	95.26	95.40	94.55	94.46	88.95
Linear	8	-	89.43	87.84	91.20	91.69	89.72	89.42	78.93
Polynomial	32	0.1	94.03	94.85	93.23	93.20	94.02	94.04	88.08
Sigmoid	16	0.01	76.61	76.01	77.26	78.19	77.09	76.60	53.23

**Table 5:** Prediction of PPIs based on protein domains or GO annotations.

	Features	Human		*B. mori*	
Indicators/%		Domains	GO Annotations	Domains	GO Annotations
Acc		93.39	93.50	90.45	88.58
Sn		95.93	96.59	88.72	90.89
Spe		91.06	90.07	92.37	86.19
Pre		90.78	91.55	92.81	87.21
F1		93.29	94.00	90.72	89.01
AUC		93.42	93.75	90.43	88.67
MCC		86.92	87.08	80.98	77.20

**Table 6:** Results of comparison with other SVM methods.

Method	Acc	Sn	Spe	MCC
	(%)			
DGO-SVM	95.52	97.73	93.47	91.14
Goktepe [43]	93.45	89.29	89.84	85.71
Zhang [44]	82.11	80.40	84.73	80.07
SP-SVM [29] ^a^	70.00	66.00	72.00	-
LDA-SVM [29] ^b^	69.00	63.00	72.00	-
PseAAC_SVM [29] ^c^	68.00	63.00	70.00	-

^a–c^ These results were taken from Table 5 of Pan’s work [29].

**Table 7:** Comparison of DGO-SVM with LR and RF.

Methods	Acc	Sn	Spe	Pre	F1	AUC	MCC
	(%)						
DGO-SVM	95.52	97.73	93.47	93.45	95.56	95.65	91.14
LR	93.47	96.25	90.95	90.62	93.35	93.51	87.10
RF	93.17	94.92	91.52	91.39	93.12	93.19	86.41

**Table 8:** Statistics of the datasets for the five aquatic crustaceans.

Organisms	Protein	Domain	Original GO	Level 4 GO	Positive PPIs	Positive PPIs (Score > 900)
*P. vannamei*	14,478	4850	3258	938	3,418,206	109,096
*A. vulgare*	11,011	4743	2811	943	2,885,527	93,932
*D. pulex*	15,113	4895	5951	1430	8,430,684	169,060
*D. magna*	11,142	4275	3389	843	3,854,957	147,805
*T. californicus*	12,634	4904	3257	965	3,257,157	128,080

**Table 9:** Prediction results of the five aquatic crustaceans.

Organisms	Acc (%)	Sn (%)	Spe (%)	Pre (%)	F1 (%)	AUC (%)	MCC (%)
*P. vannamei*	93.92	93.00	94.88	94.99	93.98	87.86	93.92
*A. vulgare*	93.28	92.42	94.19	94.31	93.35	86.59	93.28
*D. pulex*	96.51	96.14	96.88	96.91	96.52	93.02	96.51
*D. magna*	93.73	92.77	94.74	94.86	93.80	87.49	93.73
*T. californicus*	93.19	92.37	94.05	94.17	93.26	86.40	93.19

**Table 10:** The score distribution of positive and novel PPIs in *E. sinensis*.

Score	Positive PPIs	Novel Protein-Related PPIs
>0.5	7,243,597	35,604
>0.6	5,257,263	25,991
>0.7	3,588,005	16,420
>0.8	2,169,889	9343
>0.9	947,893	2938

## Data Availability

The data of *B. mori* and five aquatic crustaceans during the current study are available in STRING database (https://cn.string-db.org, accessed on 12 August 2021) and Uniprot database (https://www.uniprot.org, accessed on 25 June 2021). The taxon-Ids of the species are: 7091, 6689, 13347, 6669, 35525 and 6832. The data of humans are available from the corresponding author upon reasonable request. The genome data of *E. sinensis* were obtained from BioProject database (https://www.ncbi.nlm.nih.gov/bioproject/, accessed on 20 August 2021) under the accession number PRJNA555707 [39]. All data generated or analyzed during this study are included in the Supplementary Files.

## Supplementary Materials

The following supporting information can be downloaded at: https://www.mdpi.com/article/10.3390/cimb46070436/s1, Supplementary File S1: The positive and negative PPIs of *B. mori* and five aquatic crustaceans. There are six tables in this file. Table S1 is the positive and negative PPIs of *B. mori*. Table S2 is the positive and negative PPIs of *P. vannamei*. Table S3 is the positive and negative PPIs of *A. vulgare*. Table S4 is the positive and negative PPIs of *D. pulex*. Table S5 is the positive and negative PPIs of *D. magna*. Table S6 is the positive and negative PPIs of *T. californicus*. Supplementary File S2 The GO annotations in each GO level. Supplementary File S3 The PIN of *E. sinensis*. Supplementary File S4 Novel-protein-related PPIs in *E. sinensis* PIN.

## Abbreviations

PPI	Protein–protein Interaction
PIN	Protein–protein Interaction Network
Acc	Accuracy
Sn	Sensitivity
Spe	Specificity
Pre	Precision
AUC	Area Under Curve
MCC	Matthews Correlation Coefficient
LR	Logistic Regression
DT	Decision Trees
RF	Random Forest

## References

[1] Li J., Gou Y., Yang J., Zhao L., Wang B., Hao T., Sun J. (2022). Genome-scale metabolic network model of Eriocheir sinensis icrab4665 and nutritional requirement analysis. BMC Genom..

[2] Yu H., Braun P., Yildirim M.A., Lemmens I., Venkatesan K., Sahalie J., Hirozane-Kishikawa T., Gebreab F., Li N., Simonis N. (2008). High-quality binary protein interaction map of the yeast interactome network. Science.

[3] Geisler-Lee J., O’Toole N., Ammar R., Provart N.J., Millar A.H., Geisler M. (2007). A predicted interactome for Arabidopsis. Plant Physiol..

[4] Schwikowski B., Uetz P., Fields S. (2000). A network of protein-protein interactions in yeast. Nat. Biotechnol..

[5] Rual J.F., Venkatesan K., Hao T., Hirozane-Kishikawa T., Dricot A., Li N., Berriz G.F., Gibbons F.D., Dreze M., Ayivi-Guedehoussou N. (2005). Towards a proteome-scale map of the human protein-protein interaction network. Nature.

[6] Hao T., Peng W., Wang Q., Wang B., Sun J. (2016). Reconstruction and Application of Protein-Protein Interaction Network. Int. J. Mol. Sci..

[7] Low T.Y., Syafruddin S.E., Mohtar M.A., Vellaichamy A., NS A.R., Pung Y.F., Tan C.S.H. (2021). Recent progress in mass spectrometry-based strategies for elucidating protein-protein interactions. Cell. Mol. Life Sci..

[8] Elhabashy H., Merino F., Alva V., Kohlbacher O., Lupas A.N. (2022). Exploring protein-protein interactions at the proteome level. Structure.

[9] Pastrello C., Pasini E., Kotlyar M., Otasek D., Wong S., Sangrar W., Rahmati S., Jurisica I. (2014). Integration, visualization and analysis of human interactome. Biochem. Biophys. Res. Commun..

[10] Ito T., Chiba T., Ozawa R., Yoshida M., Hattori M., Sakaki Y. (2001). A comprehensive two-hybrid analysis to explore the yeast protein interactome. Proc. Natl. Acad. Sci. USA.

[11] Giot L., Bader J.S., Brouwer C., Chaudhuri A., Kuang B., Li Y., Hao Y.L., Ooi C.E., Godwin B., Vitols E. (2003). A protein interaction map of Drosophila melanogaster. Science.

[12] Huang X.T., Zhu Y., Chan L.L., Zhao Z., Yan H. (2016). An integrative *C. elegans* protein-protein interaction network with reliability assessment based on a probabilistic graphical model. Mol. Biosyst..

[13] Mrowka R., Patzak A., Herzel H. (2001). Is there a bias in proteome research?. Genome Res..

[14] Lalonde S., Ehrhardt D.W., Loque D., Chen J., Rhee S.Y., Frommer W.B. (2008). Molecular and cellular approaches for the detection of protein-protein interactions: Latest techniques and current limitations. Plant J..

[15] Piehler J. (2005). New methodologies for measuring protein interactions in vivo and in vitro. Curr. Opin. Struct. Biol..

[16] Hao T., Gou Y., Li J., Wang B., Zhang Y., Sun J. Construction of Eriocheir sinensis Protein-protein Interaction Network and Extraction of Molting Sub-network. Proceedings of the 12th International Conference on Bioscience, Biochemistry and Bioinformatics (icbbb2022).

[17] Gemovic B., Sumonja N., Davidovic R., Perovic V., Veljkovic N. (2019). Mapping of Protein-Protein Interactions: Web-Based Resources for Revealing Interactomes. Curr. Med. Chem..

[18] Bock J.R., Gough D.A. (2001). Predicting protein—Protein interactions from primary structure. Bioinformatics.

[19] Eid F.E., ElHefnawi M., Heath L.S. (2016). De Novo: Virus-host sequence-based protein-protein interaction prediction. Bioinformatics.

[20] Ivarsson Y., Jemth P. (2019). Affinity and specificity of motif-based protein-protein interactions. Curr. Opin. Struct. Biol..

[21] Chou K.C. (2011). Some remarks on protein attribute prediction and pseudo amino acid composition. J. Theor. Biol..

[22] Davey N.E., Cyert M.S., Moses A.M. (2015). Short linear motifs—Ex nihilo evolution of protein regulation. Cell Commun. Signal. CCS.

[23] Vogel C., Bashton M., Kerrison N.D., Chothia C., Teichmann S.A. (2004). Structure, function and evolution of multidomain proteins. Curr. Opin. Struct. Biol..

[24] Heinemann U., Schuetz A. (2019). Structural Features of Tight-Junction Proteins. Int. J. Mol. Sci..

[25] Riley R., Lee C., Sabatti C., Eisenberg D. (2005). Inferring protein domain interactions from databases of interacting proteins. Genome Biol..

[26] Binny Priya S., Saha S., Anishetty R., Anishetty S. (2013). A matrix based algorithm for Protein-Protein Interaction prediction using Domain-Domain Associations. J. Theor. Biol..

[27] Hayashida M., Kamada M., Song J., Akutsu T. (2011). Conditional random field approach to prediction of protein-protein interactions using domain information. BMC Syst. Biol..

[28] Singhal M., Resat H. (2007). A domain-based approach to predict protein-protein interactions. BMC Bioinform..

[29] Pan X.Y., Zhang Y.N., Shen H.B. (2010). Large-scale prediction of human protein-protein interactions from amino acid sequence based on latent topic features. J. Proteome Res..

[30] Huang S., Cai N., Pacheco P.P., Narrandes S., Wang Y., Xu W. (2018). Applications of Support Vector Machine (SVM) Learning in Cancer Genomics. Cancer Genom. Proteom..

[31] Huang M.W., Chen C.W., Lin W.C., Ke S.W., Tsai C.F. (2017). SVM and SVM Ensembles in Breast Cancer Prediction. PLoS ONE.

[32] Zhang M., Su Q., Lu Y., Zhao M., Niu B. (2017). Application of Machine Learning Approaches for Protein-protein Interactions Prediction. Med. Chem..

[33] Yang C., Ren J., Li B., Jin C., Ma C., Cheng C., Sun Y., Shi X. (2019). Identification of gene biomarkers in patients with postmenopausal osteoporosis. Mol. Med. Rep..

[34] Zhang Y., Ni J., Gao Y. (2022). RF-SVM: Identification of DNA-binding proteins based on comprehensive feature representation methods and support vector machine. Proteins.

[35] Li Q., Rajagopalan C., Clifford G.D. (2014). A machine learning approach to multi-level ECG signal quality classification. Comput. Methods Programs Biomed..

[36] Sahoo S.S., Kobow K., Zhang J., Buchhalter J., Dayyani M., Upadhyaya D.P., Prantzalos K., Bhattacharjee M., Blumcke I., Wiebe S. (2022). Ontology-based feature engineering in machine learning workflows for heterogeneous epilepsy patient records. Sci. Rep..

[37] Wang C., Wang X., Jing X., Yokoi H., Huang W., Zhu M., Chen S., Li G. (2022). Towards high-accuracy classifying attention-deficit/hyperactivity disorders using CNN-LSTM model. J. Neural Eng..

[38] Crawford J., Chikina M., Greene C.S. (2024). Optimizer’s dilemma: Optimization strongly influences model selection in transcriptomic prediction. Bioinform. Adv..

[39] Tang B., Wang Z., Liu Q., Zhang H., Jiang S., Li X., Sun Y., Sha Z., Jiang H., Wu X. (2019). High-Quality Genome Assembly of Eriocheir japonica sinensis Reveals Its Unique Genome Evolution. Front. Genet..

[40] Chen C., Chen H., Zhang Y., Thomas H.R., Frank M.H., He Y., Xia R. (2020). TBtools: An Integrative Toolkit Developed for Interactive Analyses of Big Biological Data. Mol. Plant.

[41] Zhang S.W., Hao L.Y., Zhang T.H. (2014). Prediction of protein-protein interaction with pairwise kernel support vector machine. Int. J. Mol. Sci..

[42] Li X., Yang L., Zhang X., Jiao X. (2019). Prediction of Protein-Protein Interactions Based on Domain. Comput. Math. Methods Med..

[43] Göktepe Y.E., Kodaz H. (2018). Prediction of Protein-Protein Interactions Using An Effective Sequence Based Combined Method. Neurocomputing.

[44] Zhang X., Jiao X., Song J., Chang S. (2016). Prediction of human protein-protein interaction by a domain-based approach. J. Theor. Biol..

[45] Martin S., Roe D., Faulon J.L. (2005). Predicting protein-protein interactions using signature products. Bioinformatics.

[46] Shen H.B., Chou K.C. (2008). PseAAC: A flexible web server for generating various kinds of protein pseudo amino acid composition. Anal. Biochem..

[47] Tang T., Zhang X., Liu Y., Peng H., Zheng B., Yin Y., Zeng X. (2023). Machine learning on protein-protein interaction prediction: Models, challenges and trends. Brief. Bioinform..

[48] Lin M., Zhou X., Shen X., Mao C., Chen X. (2011). The predicted Arabidopsis interactome resource and network topology-based systems biology analyses. Plant Cell.

[49] Camon E., Magrane M., Barrell D., Binns D., Fleischmann W., Kersey P., Mulder N., Oinn T., Maslen J., Cox A. (2003). The Gene Ontology Annotation (GOA) project: Implementation of GO in SWISS-PROT, TrEMBL, and InterPro. Genome Res..

[50] Singh R., Devkota K., Sledzieski S., Berger B., Cowen L. (2022). Topsy-Turvy: Integrating a global view into sequence-based PPI prediction. Bioinformatics.

